# Aureobasidium Melanogenum as an Uncommon Pathogen Causing Skin and Soft Tissue Infection: A Case Report and Literature Review

**DOI:** 10.7759/cureus.87056

**Published:** 2025-06-30

**Authors:** Gopi Patel, Shelly Sclater, Pooja Gurram, Sai Chintalapati

**Affiliations:** 1 Infectious Diseases, University of Florida, Gainesville, USA

**Keywords:** aureobasidium melanogenum, immunocompetent patient, immunocompromised hosts, opportunistic infections, skin and soft tissue infection

## Abstract

*Aureobasidium melanogenum* is an emerging pathogen of growing importance in medical literature. It is a dematiaceous fungus with a propensity to cause opportunistic infections in immunocompromised hosts. However, we present a case of an immunocompetent patient who developed a skin and soft-tissue infection caused by *A. melanogenum*. The pathogen was identified at a reference laboratory at the University of Texas (UT), San Antonio, Texas, using phenotypic and DNA sequencing techniques. The patient was successfully treated with a two-week course of intravenous micafungin. This case highlights the need to recognize such emerging organisms as potential causes for common community-acquired infections requiring prompt treatment rather than considering them as potential environmental contaminants.

## Introduction

Skin and soft tissue infections (SSTIs) are commonly seen as infectious disease entities, and we often use a syndromic approach to treatment, typically without making a microbiological diagnosis. However, occasionally the symptoms do not respond to such therapy, or the patient’s history and epidemiology urge us to consider atypical causes, which need more tailored antimicrobial treatment. *Aureobasidium melanogenum* (*A. melanogenum*) is an omnipresent dematiaceous fungus commonly found in the environment, including soil, freshwater, decaying organic matter, and indoor habitats [1]. It is a member of the *Aureobasidium* genus, characterized by its dimorphic nature, transitioning between yeast-like and filamentous hyphal forms depending on environmental conditions [2]. With the ability to replicate at human body temperatures, *A. melanogenum* has garnered attention as an infrequent opportunistic pathogen, particularly prone to infecting immunocompromised patients [3-5]. Implicated in cases of fungemia, *A. melanogenum* often enters the body through traumatic inoculation [6]. Clinical syndromes associated with *A. melanogenum* include cutaneous, ocular, catheter-related, pulmonary, and peritoneal infections [7]. However, despite its recognized pathogenicity, instances of *A. melanogenum* infections remain rare, and standardized diagnostic protocols and treatment guidelines have yet to be established. In this report, we present a case of patellar osteomyelitis complicated by a concurrent *A. melanogenum *skin and soft tissue infection.

## Case presentation

**Figure 1:**
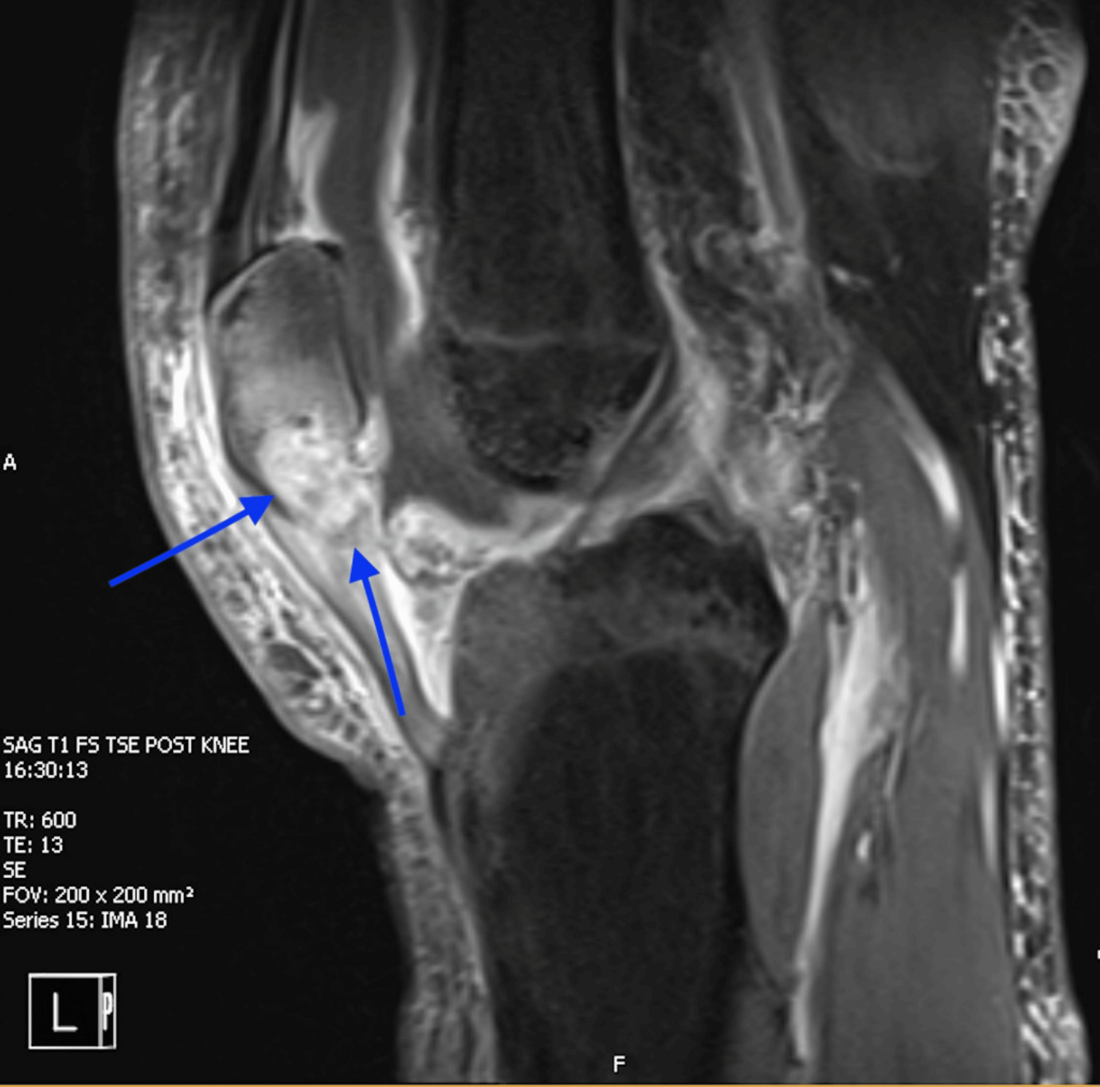
MRI of the knee post-contrast fat suppression T1 sequence showing a sagittal section demonstrating patellar osteomyelitis with synovial thickening

A 25-year-old plumber with no known comorbidities sustained a work-related injury in August 2023, when he accidentally stabbed himself in the medial left knee with a screwdriver while working under a sink during his day job as a plumber. He received a short course of oral antibiotics with minimal improvement. The wound did not heal fully, and he reported intermittent left knee pain localized over the patella, redness, and swelling, accompanied by chills and fatigue for approximately three months prior to presentation to our hospital in December 2023. On presentation, he was afebrile, hemodynamically stable, and not acutely ill-appearing. His physical examination revealed mild edema, tenderness, and warmth over the left knee. His laboratory tests showed no leukocytosis, and he had normal renal and liver function results. Imaging with MRI revealed osteomyelitis of the left inferior patella. He received empiric broad-spectrum antibiotics (vancomycin and cefepime) and underwent irrigation and debridement (Figure 1).

Initial operative bone cultures remained negative, and he was discharged home with empiric broad-spectrum parenteral antibiotics. The pathology report confirmed chronic osteomyelitis. Intraoperative bone cultures subsequently grew *Mycobacterium abscessus subsp. bolletii *(*M. abscessus*), for which treatment was switched to IV amikacin, imipenem, and tigecycline in January 2024. Three days later, the patient woke up with clear, non-odorous purulent drainage from his wound and was advised to return to the hospital. Upon readmission, he was afebrile with stable vital signs. Repeat irrigation and debridement were done. Intraoperative findings revealed features of soft tissue infection with sparing of the knee joint and patella. Soft tissue fungal cultures revealed yeast growth, later identified as *A. melanogenum*, along with the growth of previously identified *M. abscessus* in acid-fast bacilli (AFB) cultures and fungal cultures. HIV screening results were negative. The patient was subsequently treated with IV micafungin for two weeks. Since micafungin has adequate soft tissue penetration [8] and the data on treatment for *A. melanogenum* is sparse, we chose micafungin as opposed to the more toxic amphotericin. We wanted to avoid the potential for worse nephrotoxicity with the simultaneous use of amphotericin and amikacin. Additionally, he completed an intensive course of parenteral amikacin, tigecycline, and imipenem for eight weeks, followed by an oral regimen of omadacycline and clofazimine for an additional four months. On follow-up, his wounds healed, and he returned to work.

## Discussion

*A. melanogenum* is a ubiquitous, dematiaceous fungus that is a rare cause of opportunistic infections in humans [5]. It is a dimorphic fungus, with both yeast and hyphal forms depending on environmental conditions, with yeast forms often confused with Candida species. Clinically, it has been implicated in skin and soft tissue infections and fungemia associated with intravenous catheters [5,6,9]. Medical literature on infections caused by *Aureobasidium* species is scarce, with limited guidance on its diagnosis and treatment. In this report, we present a detailed case of an otherwise healthy 25-year-old patient who experienced an SSTI caused by *A. melanogenum*, which was treated with a two-week course of IV micafungin, shedding light on potential management options for *Auerobasidium* infections. Given the ubiquitous nature of *A. melanogenum* in the environment, we postulate that the patient’s initial knee injury with the screwdriver may have presented a route for traumatic inoculation, given his occupational exposure to moist and wet surfaces, which could have contaminated his tools. The patient has no prior history of infectious illnesses or similar family history suspicious of a primary immunocompromising condition. His HIV testing was negative, and he does not have diabetes mellitus. Comparative studies have shown that *A. melanogenum* exhibits higher pathogenicity compared to other black yeasts, which may explain its ability to cause infection following environmental exposure [10]. While nosocomial spread has been reported, this is less likely in our patient since this organism has not been isolated previously in such settings in our hospital, and he had a limited hospital stay perioperatively [11]. We suspect the recent and repeated exposure to broad-spectrum antibiotics likely disrupted local flora and unmasked a pre-existing fungal pathogen. Given that the patient’s symptoms did not improve with the ongoing antibiotic regimen, we considered the fungal infection to be a true pathogen and treated accordingly.

Previous reports of *A. melanogenum* infection are summarized in Table 1. 

**Table 1:** Summary of previous reports CVC: Central Venous Catheter

Patient demographics	Clinical Syndromes	Treatment Approach	Treatment Duration	Outcome	Underlying Comorbidities
20-year-old man [6]	A. melanogenum bloodstream infection	CVC removal, Initial resistance with Micafungin, switched to Amphotericin B	Six days	Successful resolution	Chronic neurological impairment secondary to cerebral injury
30-week-old infant [4]	A. melanogenum bloodstream infection	Initial resistance to fluconazole delayed initiation of Amphotericin B therapy. Catheter not removed.	Nine days	Death (multi organ dysfunction syndrome)	30-week preterm birth Very low birth weight Maternal gestational diabetes
28-year-old man [11]	Disseminated nosocomial A. melanogenum infection	IV fluconazole	Seven weeks	Successful resolution	Severe polytrauma
11-year-old boy [9]	A. melanogenum bloodstream infection	Amphotericin B	Two weeks	Successful resolution	Lymphopenia and intestinal lymphangiectasia

Diagnostics

Fungal specimens from our patient were identified as *A. melanogenum* by combined phenotypic characterization and DNA sequencing of the target’s internal transcribed spacer (ITS) and D1/D2 domains of ribosomal ribonucleic acid (rRNA). The testing was performed at a reference Fungus Testing Laboratory, UT San Antonio, TX. Previous reports of *A. melanogenum* have noted the challenges with traditional diagnostic methods for opportunistic fungal infections. As noted by Yamamoto et al. and Samaddar and Sharma et al., the pathogen can be easily confused with *Candida* in the early stages of fungal culture, as both exhibit similar phenotypic characteristics [4,6]. Additionally, diagnostic measures such as VITEK-2 and Matrix-Assisted Laser Desorption/Ionization Time-of-Flight (MALDI-TOF) failed to identify the correct organism in their report and yielded different results [4,6]. Molecular diagnostic methods proved to be successful in identifying *A. melanogenum* in our patients and paved the way for successful therapy. Literature has indicated that sequencing of the ITS and D1/D2 region is a rapid, accurate, and reliable alternative to conventional diagnostic methods for opportunistic yeast infections [12]. For best practices, researchers suggest a two-step procedure involving traditional methods for microscopic morphology and DNA sequence analysis [12], a topic that should be explored in future studies. While ITS and D1/D2 sequencing proved effective in this case, implementation in routine clinical settings may be limited by availability and high costs. This highlights the need for more alternatives to sequencing-based diagnostics that are cost-effective, rapid, and established for use in clinical microbiology laboratories.

Antifungal susceptibilities 

The yeast form grew on fungal cultures within three days, but the final identification and susceptibilities were only available to us two weeks later. The patient was empirically treated with an echinocandin and clinically improved by the time we had the results. Antifungal susceptibilities are shown in Table 2. Fluconazole had the highest minimum inhibitory concentration (MIC) at 16 μg/mL. This finding is consistent with past reports of *A. melanogenum* infection and fluconazole resistance. Currently, there are no CLSI interpretive criteria for *A. melanogenum* and no standard guidelines for the treatment approach.

In the case of a neonate with *A. melanogenum* infection, researchers found substantial fluconazole resistance at 32 ug/mL [4]. The lack of effective therapy, combined with a delayed start to amphotericin B, resulted in the patient’s eventual death. Additionally, in a chronically ill pediatric patient with *A. melongenum*, fluconazole MIC was notably high at 64 ug/ml [9].

The European Society of Clinical Microbiology and Infectious Diseases/European Confederation of Medical Mycology recommends amphotericin B and catheter removal for *Aureobasidium* bloodstream infections [4]. As shown in Table 2, Yamamoto et al. and Shier et al. indicate successful fungal infection resolution after appropriate amphotericin B therapy [6,9]. To the best of our knowledge, this is the first report to show successful resolution of *A. melanogenum* with micafungin therapy. Given the substantial risk of fluconazole resistance in *A. melanogenum* infections and the toxicities associated with amphotericin B, micafungin should be further explored as a treatment option. Interestingly, clinical isolates of *Aureobasidium* have been found to be more sensitive to fungicides than environmental isolates, suggesting that standard antifungal therapies may be effective in clinical settings [13].

**Table 2:** Current therapies and their susceptibility

Drug	Susceptibility Result (ug/ml)
Amphotericin B	0.03
Posaconazole	0.03
Micafungin	2
5 Fluorocytosine	8
Fluconazole	16

## Conclusions

*A. melanogenum* is a rare but emerging cause of not only opportunistic fungal infections in immunocompromised hosts but can also cause common community-acquired infections such as SSTIs. Genomic studies have shown that *A. melanogenum* possesses unique adaptations for survival in extreme environments, including resistance to oxidative stress and the ability to metabolize a wide range of organic compounds, which may contribute to its pathogenic potential in humans. Diagnostics are limited in clinical laboratories, often leading to delayed identification and susceptibility testing. Echinocandins may be used to treat less severe infections. Further research is warranted to establish standardized diagnostic protocols and treatment guidelines to improve outcomes for patients affected by this emerging pathogen.
